# Rhythmic Memory Consolidation in the Hippocampus

**DOI:** 10.3389/fncir.2022.885684

**Published:** 2022-04-01

**Authors:** Miriam S. Nokia, Markku Penttonen

**Affiliations:** ^1^Department of Psychology, University of Jyväskylä, Jyväskylä, Finland; ^2^Centre for Interdisciplinary Brain Research, University of Jyväskylä, Jyväskylä, Finland

**Keywords:** electrophysiology, respiration, brain oscillations, sleep, neuronal circuits

## Abstract

Functions of the brain and body are oscillatory in nature and organized according to a logarithmic scale. Brain oscillations and bodily functions such as respiration and heartbeat appear nested within each other and coupled together either based on phase or based on phase and amplitude. This facilitates communication in wide-spread neuronal networks and probably also between the body and the brain. It is a widely accepted view, that nested electrophysiological brain oscillations involving the neocortex, thalamus, and the hippocampus form the basis of memory consolidation. This applies especially to declarative memories, that is, memories of life events, for example. Here, we present our view of hippocampal contribution to the process of memory consolidation based on the general ideas stated above and on some recent findings on the topic by us and by other research groups. We propose that in addition to the interplay between neocortical slow oscillations, spindles, and hippocampal sharp-wave ripples during sleep, there are also additional mechanisms available in the hippocampus to control memory consolidation: a rather non-oscillatory hippocampal electrophysiological phenomenon called the dentate spike might provide a means to not only consolidate but to also modify the neural representation of declarative memories. Further, we suggest that memory consolidation in the hippocampus might be in part paced by breathing. These considerations might open new possibilities for regulating memory consolidation in rest and sleep.

## Introduction

Constant fluctuations in membrane potential of cells in the brain, especially synaptic activity of neurons, produce voltage fluctuations in the extracellular space that can be detected by means of local-field potential (LFP) or electrocorticogram (ECoG) recordings invasively or electroencephalogram (EEG) and magnetoencephalogram (MEG) recordings noninvasively (Buzsaki et al., 2012). When monitoring these measures, it is obvious that the electrophysiological activity of the brain is rhythmic: slow oscillations tend to involve large neural networks and span multiple brain structures whereas fast oscillations confine to smaller neural assemblies and are limited to specific regions. The oscillations are often categorized into specific frequency bands according to the following: <0.5 Hz (ultra-slow oscillations), 0.5–1 Hz (slow oscillations), 1–3.5 Hz (delta), 3.5–8 Hz (theta), 8–12 Hz (alpha), 13–30 Hz (beta), 30–80 Hz (gamma), >80 Hz (fast oscillations).

**Figure 1:**
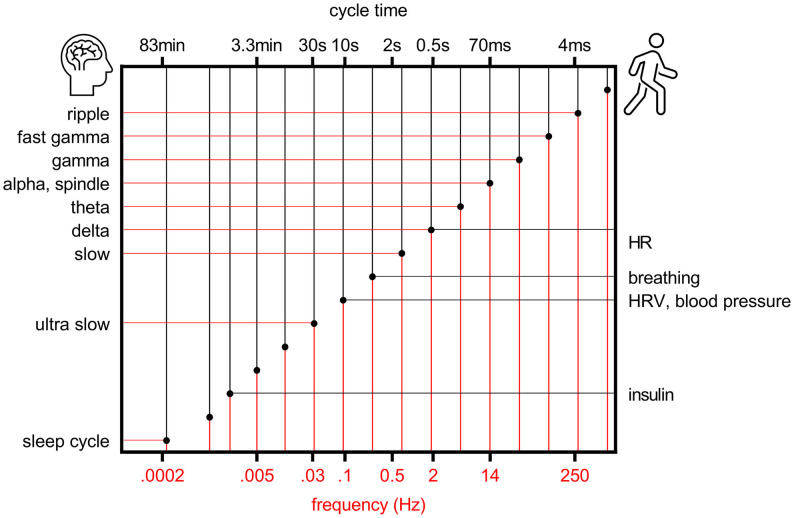
Rhythms of the brain and body follow a logarithmic scale. Organization of brain (left y-axis) and bodily (right y-axis) rhythms on a logarithmic scale, modified from Penttonen and Buzsaki (2003). HR, heart rate; HRV, heart rate variability.

Some 20 years ago it was proposed that to allow efficient and reliable communication at different temporal and spatial scales, the center frequencies of brain oscillations follow a logarithmic scale where the distance between neighboring bands is close to Neper’s number e, a real and irrational number approaching ~2.72 (Penttonen and Buzsaki, 2003; Figure 1). This ensures that signaling at neighboring frequency bands does not interfere with each other. It was further suggested that the electrophysiological oscillations of distinct frequencies are produced by separate, independent biological mechanisms in the brain and also serve distinct physiological functions (Penttonen and Buzsaki, 2003). The idea has since been further discussed and developed, demonstrating for example how similar the frequency bands of brain oscillations are in mammals from mice to humans despite the differences in the size of the nervous system (Buzsáki and Draguhn, 2004; Buzsáki et al., 2013) and by incorporating also rhythms of the body into the system of interrelated oscillations [(Klimesch, 2018), golden mean approaching ~1.62 as the base].

**Figure 2:**
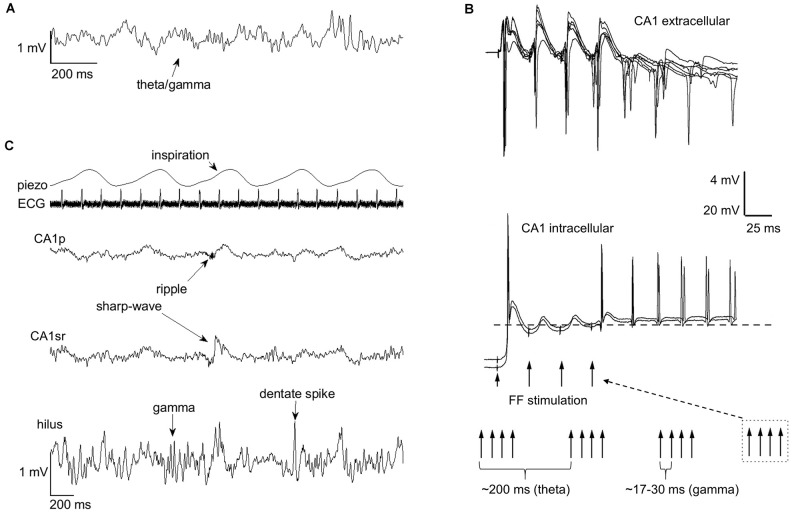
Rhythms of the brain nest within each other and within the rhythmic fluctuations of bodily states. Nesting facilitates entrainment of oscillatory activity within the hippocampus. **(A)** Phase-amplitude coupling of theta and gamma band oscillations (respectively) in the hippocampus is a well-known example of endogenous nesting of rhythmic brain activity. **(B)** The graph is modified from Mikkonen et al. (2002). Rhythmic stimulation of the fimbria fornix (FF) in urethane anesthetized Wistar rats produced entrainment of CA1 activity at the stimulation frequency, but this effect was only evident if trains of gamma-band (30–60 Hz) stimulations were conducted at theta (3–7.5 Hz) frequency, mimicking the naturally occurring theta-gamma nested oscillations in the hippocampus. In addition to entrainment, a sustained rhythmic response retaining the stimulation gamma frequency, probably dependent on the resonant properties of CA1 interneurons and pyramidal cells, is also evident. **(C)** Rhythms of the brain also seem to nest within the rhythms of the body. The occurrence of hippocampal sharp-wave ripples, gamma bursts, and dentate spikes are paced by breathing (piezo). The example traces in **(A,C)** are from a urethane-anesthetized adult male Sprague-Dawley rat. ECG, electrocardiogram; CA1p, CA1 pyramidal cell layer; CA1sr, CA1 stratum radiatum.

As explained above, brain electrophysiological oscillations are organized into separate frequency bands to avoid interference. However, oscillations at different frequency bands interact by means of phase-amplitude coupling or by phase-phase coupling, usually so that the phase of the slower oscillation modulates the amplitude or phase of the faster oscillation with n:m ratios where n and m are integer numbers. Phase-phase coupling is thought to facilitate communication by aligning the duty cycles of two oscillating neural assemblies while suppressing information flow between assemblies in which duty cycles do not overlap (Fries, 2005). An example would be the phase synchrony between distant neocortical regions in humans demonstrated in various studies and at various frequencies (Palva and Palva, 2018). A well-known example of phase-amplitude coupling in the hippocampus is that between theta phase and gamma amplitude (Lisman and Jensen, 2013; Colgin, 2015; Figure 2A). Another is the ultra-slow (0.025 Hz, cycle duration ~40 s) variation in neuronal excitability and the amplitude of beta (8–22 Hz) oscillation (Penttonen et al., 1999; see also Achermann and Borbély, 1997). This kind of link between oscillations is generally referred to as nesting, and it has been suggested to facilitate communication in neural networks (Bonnefond et al., 2017) much like the direct phase-phase coupling.

Brain oscillations emerge spontaneously but can also be entrained (Figure 2B). In humans, entrainment can be obtained by rhythmic sensory stimulation (Henao et al., 2020) or by using non-invasive methods such as transcranial magnetic stimulation (Thut et al., 2011). A straightforward means in animal models is to electrically stimulate the brain at a certain frequency to induce rhythmic fluctuations in neuronal excitability. Interestingly, stimulation of the fimbria fornix at a pattern mimicking endogenous theta/gamma-coupling entrains persistent oscillatory responses in the hippocampal CA1 (Mikkonen et al., 2002) while neither gamma nor theta stimulation alone has such an outcome. Remarkably, stimulation of just one pyramidal neuron in the CA3 is able to entrain the firing of neurons in the CA1 taken that this stimulation is timed to occur in synchrony with extracellular stimulation at the nested theta and slow frequencies (Mikkonen et al., 2006). This is to demonstrate the influence of even single neurons on the network activity of the brain, an effect that seems to directly depend on synchrony.

Entrainment can also take place in the brain endogenously, that is, without external stimulation. This might be especially prominent during non-rapid eye movement (NREM) sleep, when the brain is at a state of low synchronization: Epileptic seizures are most likely during this state in human Alzheimer’s disease (AD) patients (Horváth et al., 2017) as well as in transgenic mouse models of AD (Gureviciene et al., 2019). On the other hand, in a healthy brain, endogenous entrainment of nested oscillatory activity during sleep might facilitate memory consolidation by allowing recurrent activation of neural assemblies in wide-spread networks formed during the previous wake period.

## Slow Oscillations, Spindles, and Sharp-Wave Ripples Align to Support Memory Consolidation

Hippocampal CA1 pyramidal cells fire in sequences during awake behavior and presumably form neural representations of the experiences. During subsequent sleep, the CA1 firing sequences are replayed in condensed form (Buzsaki, 2015; Liu et al., 2019). This replay of the neural representations is evident in hippocampal LFPs as so-called sharp-wave ripples (SPW-Rs; Buzsaki, 2015). They are set forth within the hippocampus as a result of an interplay of inhibitory interneurons and excitatory pyramidal neurons of the CA2 and CA3 subregions (Csicsvari et al., 2000; Oliva et al., 2016) as well as perhaps the subiculum (Imbrosci et al., 2021). As a result, thousands of CA1 hippocampal pyramidal cells fire in synchrony which is manifested in the LFP as a burst (~100 ms) of fast oscillations (~150–250 Hz, ripple). Within the hippocampus, SPW-Rs lead to sustained synaptic facilitation between groups of CA3 and CA1 pyramidal cells potentiated during experience (Sadowski et al., 2016; see also Norimoto et al., 2018). Further, SPW-Rs are associated with signaling from the hippocampus to the neocortex *via* the subiculum (Böhm et al., 2015) and the reactivation of neocortical neural assemblies also formed during the experience (Ji and Wilson, 2007). Importantly, disrupting brain activity associated with awake (Nokia et al., 2012) or sleep (Girardeau et al., 2009) SPW-Rs hampers learning while facilitating it improves learning (Maingret et al., 2016).

SPW-Rs are most common during non-rapid eye movement sleep and occur nested to spindles (9–15 Hz) and neocortical slow oscillations (SOs, <1 Hz; Siapas and Wilson, 1998; Maingret et al., 2016; Jiang et al., 2019; Varela and Wilson, 2020). Neocortical SOs might actually drive the occurrence of spindles that then regulate the emergence of hippocampal SPW-Rs (Oyanedel et al., 2020; Peyrache and Seibt, 2020; Varela and Wilson, 2020). A study in humans recently reported directional information flow from the neocortex to the hippocampus during spindles and preceding the associated hippocampal SPW-Rs (Ngo et al., 2020). Further, neocortical SOs and spindle density correlate with memory performance (Hanert et al., 2017), and sleep spindle-contingent targeted memory reactivation by presenting auditory cues can enhance memory in humans (Antony et al., 2018). To summarize, SOs, spindles, and SPW-Rs seem to support the consolidation of memories into long-term storage (Klinzing et al., 2019).

The interplay between SOs, spindles, and SPW-Rs seems to be a textbook example of hierarchically ordered electrophysiological brain oscillations that have a distinct mechanism of generation, occur nested to each other, and that contribute to specific physiological functions (Penttonen and Buzsaki, 2003). However, some events within the brain are less oscillatory in nature, yet they still seem to have a specific mechanism of generation and a distinct physiological function. Regarding hippocampal memory consolidation, the dentate spike makes a good example.

## Dentate Spikes Possibly Enable Memory Modulation Within The Hippocampus

Dentate spikes are fast (~20–80 ms), high-amplitude (~1–2.5 mV) events evident in LFPs recorded from the hilus of the hippocampal dentate gyrus (DG) in rodents (Bragin et al., 1995; Penttonen et al., 1997; Headley et al., 2017). During dentate spikes, entorhinal neocortical activation stemming from both lateral and medial parts arrives at the DG *via* the perforant path and evokes a rapid increase in the firing rate of granule cells and interneurons (Bragin et al., 1995; Senzai and Buzsaki, 2017). This is noteworthy because granule cells fire seldom overall and are the cells that supposedly form the initial hippocampal engram (Kitamura et al., 2017) supporting reliable, orthogonal encoding of unique but similar experiences (Rolls, 2018). At the same time, dentate spikes seem to have a feed-forward inhibitory effect on CA3 (Bragin et al., 1995; Sanchez-Aguilera et al., 2021) and CA1 (Penttonen et al., 1997) pyramidal cells as they hyperpolarize and decrease firing concomitant with dentate spikes. That is, whereas SPW-Rs reflect increased firing of neurons projecting from the hippocampus to the neocortex, dentate spikes have an opposite effect resulting in a pause in hippocampal output.

Much like SPW-Rs, dentate spikes take place, especially during quiet rest and sleep. Their likelihood is increased during neocortical SO excitatory (UP) states and within a narrow time-period (~50 ms) following SPW-Rs (Headley et al., 2017) but dentate spikes mostly emerge in the absence of SPW-Rs (Bragin et al., 1995). As dentate spikes seem to exert a suppressive effect on the firing of CA1 pyramidal cells (Penttonen et al., 1997) it is not surprising that when they co-occur with SPW-Rs, the ripples are smaller in amplitude than in the absence of dentate spikes (Headley et al., 2017). In fact, dentate spikes seem to suppress the occurrence of SPW-Rs at least for a duration of 200 ms (Bragin et al., 1995). This is rather interesting as, on the other hand, input from DG granule cells *via* the mossy fibers is reported necessary for learning-related increases in CA3 awake SPW-Rs (Sasaki et al., 2018). That is, input from DG granule cells might in fact contribute to initiating SPW-Rs, except during dentate spikes, when the effect would be opposite.

Interestingly, unlike SPW-Rs, dentate spikes are not oscillatory in themselves. However, they are sometimes surrounded by a few gamma cycles in the DG [type 1 dentate spikes (Bragin et al., 1995)]. It is not entirely clear how hippocampal theta/gamma synchronization (Lisman and Jensen, 2013) relates to dentate spikes but dentate spikes are accompanied by interregional synchronization of neocortical gamma (35–100 Hz; Headley et al., 2017). Gamma-band synchronization in the hippocampo-neocortical system has long been suggested to play a role in memory formation (Axmacher et al., 2006) and might thus also involve the hippocampal dentate spike. It could be that the hippocampal gamma oscillation aids in recruiting specific granule cells to fire (de Almeida et al., 2009; Lisman and Jensen, 2013) during a (type 1) dentate spike. Together with the evidence suggesting that the DG controls SPW-R generation in the CA3/CA1 (see the end of the previous paragraph), this hints at a possible link between dentate spikes and pattern separation/completion in the DG (Leutgeb et al., 2007; Neunuebel and Knierim, 2014).

We suggest a specific function of dentate spikes in memory consolidation could be to selectively reorganize neural representations within the hippocampus, based on input from the entorhinal neocortex. Our own findings (Nokia et al., 2017; Lensu et al., 2019) imply dentate spikes as a likely candidate for merging together discrete but related neural representations during rest and sleep: Specifically, disrupting dentate spikes after training in classical conditioning of the eyeblink response hindered learning suggesting that normally dentate spikes are needed for making associations between temporally separate events, in this case, the conditioned and the unconditioned stimulus (Nokia et al., 2017). On the contrary, the same dentate spike-contingent disruption conducted after a context-object discrimination task promoted later performance, that is, memory for the two similar context-object configurations was more accurate (Lensu et al., 2019). This implies that, in our latter study, disrupting dentate spikes prevented the merging of representations of recent similar experiences. Further, promoting DG to CA3 feed-forward inhibition (also evident during dentate spikes, see above) can prevent spontaneous generalization of fear in mice over time (Guo et al., 2018). In sum, dentate spikes clearly have the potential to regulate the spontaneous reorganization of newly acquired neural representations within the hippocampus during rest. More studies are needed to better characterize the significance of dentate spikes in terms of memory consolidation.

## Respiration Might Pace Memory Consolidation in The Hippocampus and Beyond

Another angle to the organized oscillatory activity of the brain is the fact that the brain is a functionally inseparable part of the body. It has been suggested that bodily functions might organize according to a hierarchical system similar to brain oscillations (Klimesch, 2018; Figure 1). That is, the principles of facilitating communication within the brain as well as across the brain and body would essentially be scale-free, applying to oscillations differing in frequency by an order of magnitude. An interesting question is whether, during evolution, synchrony between oscillations first emerged within the bodily functions (for example respiratory-sinus arrhythmia) or within the brain or perhaps within and across the brain and body at once.

Possibly the most studied bodily rhythms affecting the brain is respiration (see for example Heck et al., 2019). In humans, MEG signals at awake, eyes-open resting state are amplitude-modulated according to the phase of breathing: Signal amplitude at delta (~2 Hz), gamma (~75 Hz), and fast (~130 Hz) frequency bands is larger near the inspiration peak and at beta (~30 Hz) band the signal amplitude is greatest during inspiration onset (Kluger and Gross, 2021). In mice, it seems that many of the hippocampal oscillations related to memory consolidation phase-lock to respiration: Specifically, a recent quite extensive report suggests SPW-Rs and dentate spikes are more likely during or right after inspiration than during expiration (Karalis and Sirota, 2022; but see also Liu et al., 2017). Our own preliminary results suggest dentate spikes, as well as dentate gyrus gamma bursts, are more abundant during inspiration than expiration also in urethane anesthetized rats (Nokia et al. under preparation) (Figure 2C). In addition to hippocampal oscillations, in mice, breathing also seems to pace prefrontal cortical activity and the interplay between the cortex and the hippocampus during sleep (Karalis and Sirota, 2022). Further, in rats, the greatest drive from respiration to wide-scale brain oscillations is obtained during awake rest by long and deep inspirations (Girin et al., 2021). To summarize, breathing might set the stage for effective memory consolidation during rest and sleep by synchronizing the oscillatory activity of the brain at large.

Further studies are needed to validate the findings reviewed above, using different species and under varying brain and bodily states. It would be interesting to know if the brain oscillations believed to support memory consolidation are governed by respiration in humans. Indeed, in awake healthy humans, nasal respiration (as opposed to oral respiration) seems to drive limbic system activity (Zelano et al., 2016) and to support memory consolidation (Arshamian et al., 2018). Specifically, when adult participants rehearsed an odor recognition memory task and then, during a 1-h delay, were forced to breathe only through the nose or the mouth, participants breathing through the nose recognized the memorized odors better compared to those breathing through the mouth (Arshamian et al., 2018). Perhaps an effective way to facilitate memory consolidation *via* breathing would be to take long and deep inspirations (Karalis and Sirota, 2022) through the nose (Girin et al., 2021).

## Discussion

To summarize, we suggest that while memory consolidation in the hippocampus is convincingly demonstrated to revolve around SPW-Rs, also dentate spikes might play a specific and perhaps complementary role (Nokia et al., 2017; Lensu et al., 2019). Further, we propose that respiration might govern memory consolidation in the hippocampus by pacing the occurrence of oscillatory phenomena (Karalis and Sirota, 2022). While there is ample evidence for the hippocampal neuronal activity related to SPW-Rs, the difficulty in recording single-unit activity from granule cells has impeded the study of dentate spike-related processing in the DG (Senzai and Buzsaki, 2017; Sanchez-Aguilera et al., 2021). However, some recent reports utilizing calcium imaging of the DG granule cells during memory tasks have yielded promising results (Pofahl et al., 2021). In the future it would be most interesting to accompany DG calcium imaging with both electrophysiology from the hippocampus as well as physiological signals from the body (respiration and heartbeat) in conjunction with a declarative memory task and subsequent sleep in freely moving rodents, to further probe the mechanisms of memory consolidation in the hippocampus.

## Data Availability Statement

The raw data supporting the conclusions of this article will be made available by the authors, without undue reservation.

## Ethics Statement

The animal study was reviewed and approved by Project Authorisation Board, Regional State Administrative Agency (licence number ESAVI/24666/2018).

## Author Contributions

MN and MP wrote the article. All authors contributed to the article and approved the submitted version.

## Conflict of Interest

The authors declare that the research was conducted in the absence of any commercial or financial relationships that could be construed as a potential conflict of interest.

## Publisher’s Note

All claims expressed in this article are solely those of the authors and do not necessarily represent those of their affiliated organizations, or those of the publisher, the editors and the reviewers. Any product that may be evaluated in this article, or claim that may be made by its manufacturer, is not guaranteed or endorsed by the publisher.
